# Fractal analysis of low attenuation clusters on computed tomography in chronic obstructive pulmonary disease

**DOI:** 10.1186/s12890-018-0714-5

**Published:** 2018-08-29

**Authors:** Naoya Tanabe, Shigeo Muro, Susumu Sato, Tsuyoshi Oguma, Atsuyasu Sato, Toyohiro Hirai

**Affiliations:** Department of Respiratory Medicine, Graduate School of Medicine, Kyoto University, 54 Kawahara-cho, Shogoin, Sakyo-ku, Kyoto, 606-8507 Japan

**Keywords:** Chronic obstructive pulmonary disease, Computed tomography, Emphysema, Fractal, Imaging, Radiography

## Abstract

**Background:**

The fractal dimension characterizing the cumulative size distribution of low attenuation area (LAA) clusters, identified with a fixed threshold such as − 950 Hounsfield Units (HU), on computed tomography (CT) sensitively detects parenchymal destruction in chronic obstructive pulmonary disease (COPD) even when the percent LAA (LAA%), a standard emphysema index, is unchanged. This study examines whether the cumulative size distribution of LAA clusters, defined with thresholds of the 15th, 25th, and 35th percentiles of a CT density histogram instead of the fixed-threshold of − 950 HU, exhibits a fractal property and whether its fractal dimension (D’15, D’25, and D’35, respectively) provides additional structural information in emphysematous lungs that is difficult to detect with the conventional − 950-HU-based fractal dimension (D950).

**Methods:**

Chest inspiratory CT scans and pulmonary functions were cross-sectionally examined in 170 COPD subjects. A proxy for the inspiration level at CT scan was obtained by dividing CT-measured total lung volume (CT-TLV) by physiologically measured total lung capacity. Moreover, long-term (> 5 years) changes in D950 and the new fractal dimensions were longitudinally evaluated in 17 current and 42 former smokers with COPD.

**Results:**

D950, but not D’15, D’25, or D’35 was weakly correlated with the proxy for the inspiration. D950, D’25, and D’35 but not D’15 correlated with LAA% and diffusion capacity. In the long-term longitudinal study, LAA% was increased and D950 and D’35 were decreased in both current and former smokers, while D’25 was decreased only in current smokers and D’15 was not changed in either group. The longitudinal changes in D’25 but not those in LAA%, D950, D’15, and D’35 were greater in current smokers than in former smokers. This greater change in D’25 in current smokers was confirmed after adjusting the change in CT-TLV and the baseline D’25.

**Conclusions:**

D’25 reflects diffusion capacity in emphysematous lungs and is robust against inspiration levels during CT scans. This new fractal dimension might provide additional structural information that is difficult to detect with the conventional D950 and LAA% and allow for more sensitive evaluation of emphysema progression over time.

**Electronic supplementary material:**

The online version of this article (10.1186/s12890-018-0714-5) contains supplementary material, which is available to authorized users.

## Background

Chronic obstructive pulmonary disease (COPD) is a worldwide health problem [1]. Emphysema is a pathological feature of the parenchyma [2] that is closely associated with airflow obstruction [3], impaired diffusion capacity [4], frequent exacerbations [5], and a poor prognosis [6, 7]. In addition, this emphysematous destruction is common even in smokers without COPD [8] and is associated with an increase in their mortality [9]. However, no therapeutic agent against parenchymal destruction is currently available. Therefore, a robust and sensitive assessment of emphysema progression is required to deepen the understanding of its pathogenesis, to evaluate the effects of new therapeutic candidate drugs in pharmacological studies, and to ultimately improve the prognosis.

Computed tomography (CT) is widely used to estimate the severity of emphysema [10]. Investigators have quantified emphysema on CT using the percentage of the low attenuation area (LAA), which is defined as a region where the CT density is below a fixed threshold, such as − 950 Hounsfield units (HU), relative to the total lung area (the percentage of LAA to the total lung area [LAA%]) [11, 12] and the percentile point defined as the cut-off value in HU below which a given percentage of all voxels is distributed [13]. Both the LAA% and the percentile point method reflect emphysema on histology [14–16].

The concept of fractals was first introduced by Mandelbrot to evaluate the complexity of natural objects [17] and has been used in many research fields including morphometry of human organs and analysis of fluctuations in philological measures over time [18]. A previous report by Mishima et al. [4] applied this concept in quantitative assessment of emphysema on CT by identifying neighboring LAA pixels as a cluster and demonstrated that the cumulative size distribution of these LAA clusters has the fractal property that is characterized by the fractal dimension D. These authors suggested that a reduction in the D value can sensitively detect parenchymal destruction and an impaired diffusion capacity in early-stage COPD, even when LAA% is unchanged. This notion is further supported by a computer simulation study by Suki et al. [19], who demonstrated that enhanced mechanical force in local regions accounts for spatially heterogeneous destruction of the parenchyma characterized by a reduced fractal dimension. Since then, analysis using a combination of the D value and LAA% has made it possible to predict outcomes after lung volume reduction surgery [20], reveal an association between exacerbations and emphysema progression [21], and demonstrate the heterogeneous progression of emphysema in smokers with COPD [22].

The LAA clusters used for calculating the fractal dimension have been currently identified using a fixed threshold such as − 950 HU. However, considering that a report on the percentile point method has shown that any of the 10th to 30th percentile points can change similarly over time and reflect emphysema progression [23] and that the 15th percentile point has been widely used for evaluation of emphysema [13, 24], it is possible that when clustering neighboring pixels in which the CT density is below a given percentile of a CT density histogram, the cumulative size distribution of these “lower” attenuation clusters also exhibits a fractal property. Therefore, the present study examines the following hypothesis: (1) the cumulative size distribution of the LAA clusters defined with thresholds of the 15th, 25th, and 35th percentiles of a CT density histogram, exhibits a fractal property characterized by the fractal dimension (D’15, D’25, and D’35, respectively), and (2) the values of D’15, D’25, and D’35 are robust against variations in the inspiration level during the CT scan and may provide additional structural information in emphysematous lungs that is difficult to detect with the conventional D based on the fixed threshold.

## Methods

### Ethics

The ethics committee of Kyoto University approved this study (approval No. E182). Written informed consent was obtained from all participants.

### Subjects’ enrollment

This study is a retrospective analysis of data from a prospective observational study at Kyoto University [21, 25] and consists of 3 analyses. The inclusion criteria for this observational study were (1) a smoking history of at least 20 pack-years and (2) COPD diagnosis. The exclusion criteria were (1) alpha-1 antitrypsin deficiency, (2) other lung disease, such as bronchial asthma, interstitial pneumonia, and bronchiectasis, (3) a history of malignancy within the past 5 years, and (4) a prior history of lung surgical resection. The follow-up of the enrolled subjects would be ongoing as of April 2018 unless they withdrew the consent of the participation or stopped visiting the outpatient clinic of Kyoto University Hospital. The specific inclusion and exclusion criteria for each of the 3 analyses were described in the Online Supplementary Methods. The first analysis included 170 subjects with COPD who underwent chest CT scans and pulmonary function tests during non-exacerbating periods between April 2010 and March 2014. The second analysis included 33 subjects who were clinically stable but underwent 3 CT scans within 1 year because they required close follow-up of small abnormal shadows other than emphysema (short-term longitudinal study). The third analysis initially included 60 subjects whose smoking status was not changed between the baseline and > 5-year apart CT scans, and then their changes in CT-measured total lung volume (CT-TLV) from the baseline to follow-up scans were calculated. By assuming that a more than 20% change in CT-TLV from the baseline to the follow-up scans indicates inappropriate breath holding at either scan, 1 subject who showed a 32% reduction in CT-TLV at the follow-up scan was excluded, and 59 subjects were used in the third analysis.

### Pulmonary function test

Spirometry, lung volume, and carbon monoxide diffusing capacity using the single-breath method (D_LCO_) were measured with a Chestac-65 V (Chest MI Corp., Tokyo, Japan).

### CT acquisition

All CT images at full inspiration were obtained with an Aquilion 64 scanner (Toshiba; Tokyo, Japan). The CT scanner was calibrated routinely with air and water phantoms. Subjects were instructed to hold breath at full inspiration during a CT scan. The scanning condition was 0.5-mm collimation, a scan time of 500 milliseconds, 120 kV peak (kVp), and auto-exposure control. The reconstruction was performed with a high spatial frequency algorithm (FC56) as previously reported [21].

### CT analysis

**Fig. 1:**
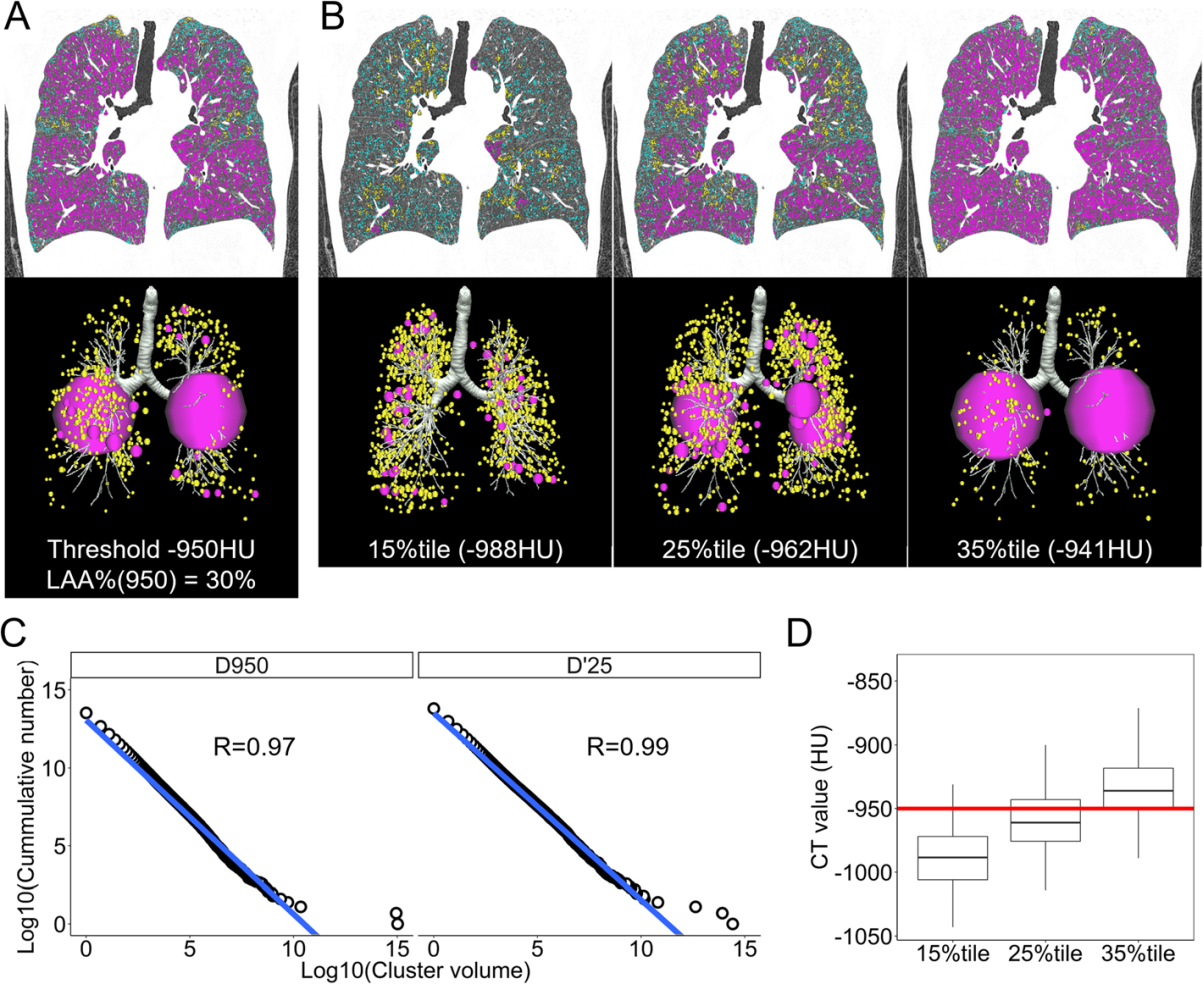
Examples of low attenuation cluster analysis using either the threshold of a fixed CT density or that of the 15th, 25th, or 35th percentile of the CT density histogram. **a** and **b** Coronal sections of CT images show low attenuation areas (LAAs) based on thresholds of (**a**) fixed − 950 Hounsfield Units (HU) and (**b**) the 15th, 25th, and 35th percentiles of a histogram of CT values from all pixels in whole lungs. In 3-dimensional representation, the LAA clusters were expressed as spheres with volumes equivalent to those of actual clusters. Pink, yellow, and blue regions indicate large (> 500 mm^3^), moderate (> 50 and < 499 mm^3^), and small (< 50 mm^3^) clusters, respectively. Small clusters were omitted in the 3-dimensional representation. **c** The log-transformed volumes of the LAA clusters and the log-transformed cumulative counts of the clusters larger than the given volume are plotted. This plot is well characterized by a linear regression. The fractal dimension D was obtained as an absolute slope of the regression line. D950 and D’25 indicate the fractal D characterizing LAA clusters based on the fixed − 950 HU and the 25th percentile, respectively. **d** Distributions of the 15th, 25th, and 35th percentiles of the CT values used in analysis 1 (*n* = 170) are shown

Using a SYNAPSE VINCENT volume analyzer (FUJIFILM Medical, Tokyo, Japan), the airway tree and thoracic cage were removed from the original CT images, and lung fields defined as regions < − 200 HU were segmented. The segmented lung fields were exported as DICOM files and were analyzed with custom-made software. LAAs were identified using a threshold of − 950 HU and LAA% was measured as previously reported [3, 21, 26, 27]. In addition, an LAA cluster was identified 3-dimensionally by connecting neighboring voxels for which the CT value was lower than a given threshold (Fig. 1 and Additional file 1: Figure S1). The threshold used in this study included not only a conventional fixed − 950 HU but also the 15th, 25th, and 35th percentiles of a histogram of CT values from all pixels of the lungs (Fig. 1a and b, and Additional file 1: Figure S1A and B). For each of these thresholds (− 950 HU and the 15th, 25th, and 35th percentiles), the cumulative frequency distribution of the volumes of these LAA clusters was analyzed by plotting log-transformed volumes of the clusters on the x-axis and log-transformed cumulative counts of the clusters that were larger than the given volume on the y-axis (Fig. 1c). Linear regression was performed in this log-log plot, and the coefficient of correlation (R) was calculated to evaluate the goodness of fit for the regression (Additional file 1: Figure S1D). A better R value indicates that the cumulative number Y of the clusters larger than the volume X is more accurately described as follows: Y=K · X^-D^, which is mathematically equivalent to the fractal property [4, 17]. In this equation, the value of D is the fractal dimension and is calculated as the absolute slope of the regression line on the log-log plot. In this study, each D value was based on the LAA clusters from − 950 HU and the 15th, 25th, and 35th percentiles of a CT density histogram. These values are referred to as D950, D’15, D’25, and D’35, respectively.

### Statistics

The data are expressed as the mean ± SD. The Pearson correlation test was used to evaluate associations of CT measures with pulmonary function and a proxy for inspiration during a CT scan that was obtained by dividing the CT-based total lung volume (CT-TLV) by the physiologically measured total lung capacity (TLC) [28]. Paired and non-paired t-tests were used for group comparisons, and the Holm correction was used when necessary. Multivariate linear regression analysis that used CT indexes as independent variables and each value of pulmonary function as the dependent variable was performed. In the short-term longitudinal study, the coefficient of variation (CV%) of each CT index for 3 measurements within 1 year was calculated as follows: 100 · SD / mean. Statistical analysis was performed with the R program [29]. A *p*-value less than 0.05 was considered statistically significant.

## Results

### Analysis 1 (cross-sectional data)

Table 1 shows the demographic data of the study subjects in the analysis of the cross-sectional data (*n* = 170). Among them, 144 were considered to have the better-quality CT scan, as their proxy for inspiration was more than 0.8 and less than 1.2. The distributions of the 15th, 25th, and 35th percentiles are shown in Fig. 1d.

**Table 1:** Demographics of the subjects for analysis 1

	Overall	Better-quality scans [Proxy for inspiration > 0.8 and < 1.2]
No. of subjects	170	144
Age	71 ± 9	72 ± 8
Sex (Male: Female)	162: 8	139: 5
Body mass index	22 ± 3	22 ± 3
Pack-years	61 ± 35	62 ± 36
Pulmonary function		
FEV_1_ (% predicted)	61 ± 21	60 ± 21
TLC (L)	5.8 ± 1.0	5.8 ± 1.0
RV/TLC (%)	41 ± 8	42 ± 8
D_LCO_ (% predicted)	56 ± 19	53 ± 19
D_LCO_/V_A_	2.9 ± 1.1	2.8 ± 1.0
CT		
LAA% (%)	29 ± 9	30 ± 9
CT-TLV (L)	5.2 ± 1.0	5.4 ± 1.0
Proxy for inspiration	0.91 ± 0.12	0.93 ± 0.08
15th percentile (HU)	−988 ± 23	− 991 ± 22
25th percentile (HU)	− 959 ± 24	− 962 ± 22
35th percentile (HU)	− 935 ± 24	− 938 ± 23

Data are expressed as the mean ± SD. A proxy for inspiration indicates the degree of the inspiration level during a CT scan that was obtained by dividing the total lung volume measured on CT (CT-TLV) by the total lung capacity (TLC) measured in pulmonary function tests. An appropriate quality CT scan was considered when the proxy for inspiration was more than 0.8 and less than 1.2. *FEV*_*1*_ Forced expiratory volume in 1 s, *RV/TLC* Residual volume / total lung capacity, *D*_*LCO*_ Diffusion capacity, *V*_*A*_ Alveolar ventilation, *LAA%* Percent low attenuation area less than − 950 Hounsfield units (HU)

**Fig. 2:**
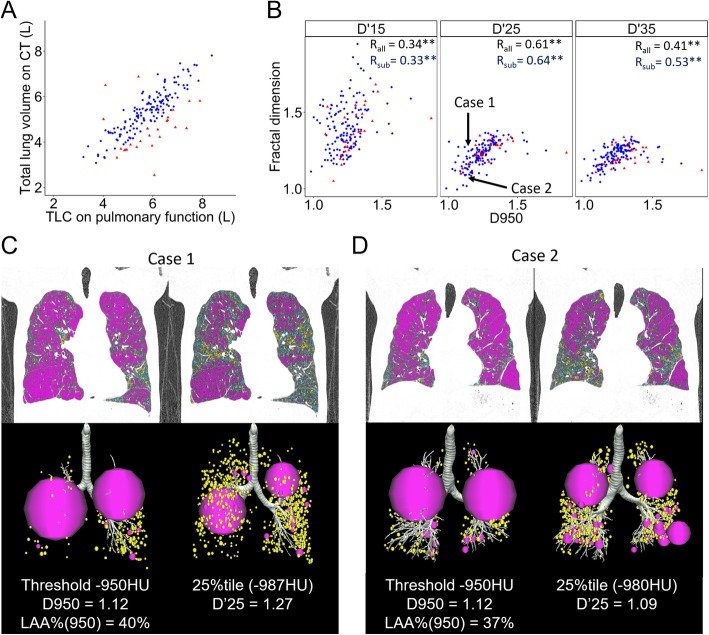
Comparisons of new fractal dimensions to the conventional fractal dimension. **a** The relationship between the CT-derived total lung volume (CT-TLV) and physiologically-measured total lung capacity (TLC). Blue dots indicate cases with better-quality scans defined as those with ratios of CT-TLV to TLC (the proxy for inspiration) > 0.8 and < 1.2. **b** Correlation between D based on thresholds of − 950 HU (D950) and the 15th, 25th, and 35th percentiles of a CT density histogram (D’15, D’25, and D’35) are shown. R_all_ and R_sub_ indicate coefficient correlations for the analyses of all scans (*n* = 170) and the better-quality scans (*n* = 144), respectively; ** *p* < 0.01. **c** and (**d**) show examples of 2 cases, both of which had similar D950 but different D’25 values. Large (> 500 mm^3^, pink) and moderate (> 50 and < 499 mm^3^, yellow) low attenuation clusters are expressed as spheres with volumes equivalent to that of actual clusters. Small (< 50 mm^3,^ blue) clusters are shown only in the coronal CT images. When using a fixed threshold of − 950 HU, both cases showed 2 large clusters and many small clusters similarly. In contrast, when using a threshold of the 25th percentile of the CT value histogram, case 2 (**d**) showed several (4 or more) relatively larger clusters and other small clusters, whereas case 1 (**c**) still showed 2 larger clusters and many small clusters

In Fig. 2a, which shows relationship between CT-TLV and TLC, blue dots indicate cases with better-quality scans (the proxies for inspiration were > 0.8 and < 1.2). Figure 2b shows that D950 was correlated with D’15, D’25 and D’35 in both the analysis of all scans (*n* = 170) and the subanalysis of the better-quality scans (*n* = 144). Figure 2c and d shows examples of 2 cases with similar D950 and different D’25 values. When the low attenuation clusters were defined with a fixed threshold of − 950 HU, both cases showed 2 large clusters (pink spheres) and many small clusters (yellow spheres) similarly. In contrast, when the low attenuation clusters were defined with a threshold of the 25 percentile of the CT value histogram, case 2 showed several (4 or more) relatively larger clusters and other small clusters, whereas case 1 still showed 2 larger clusters and many small clusters. The D’25 value was lower in case 2 than in case 1.

**Fig. 3:**
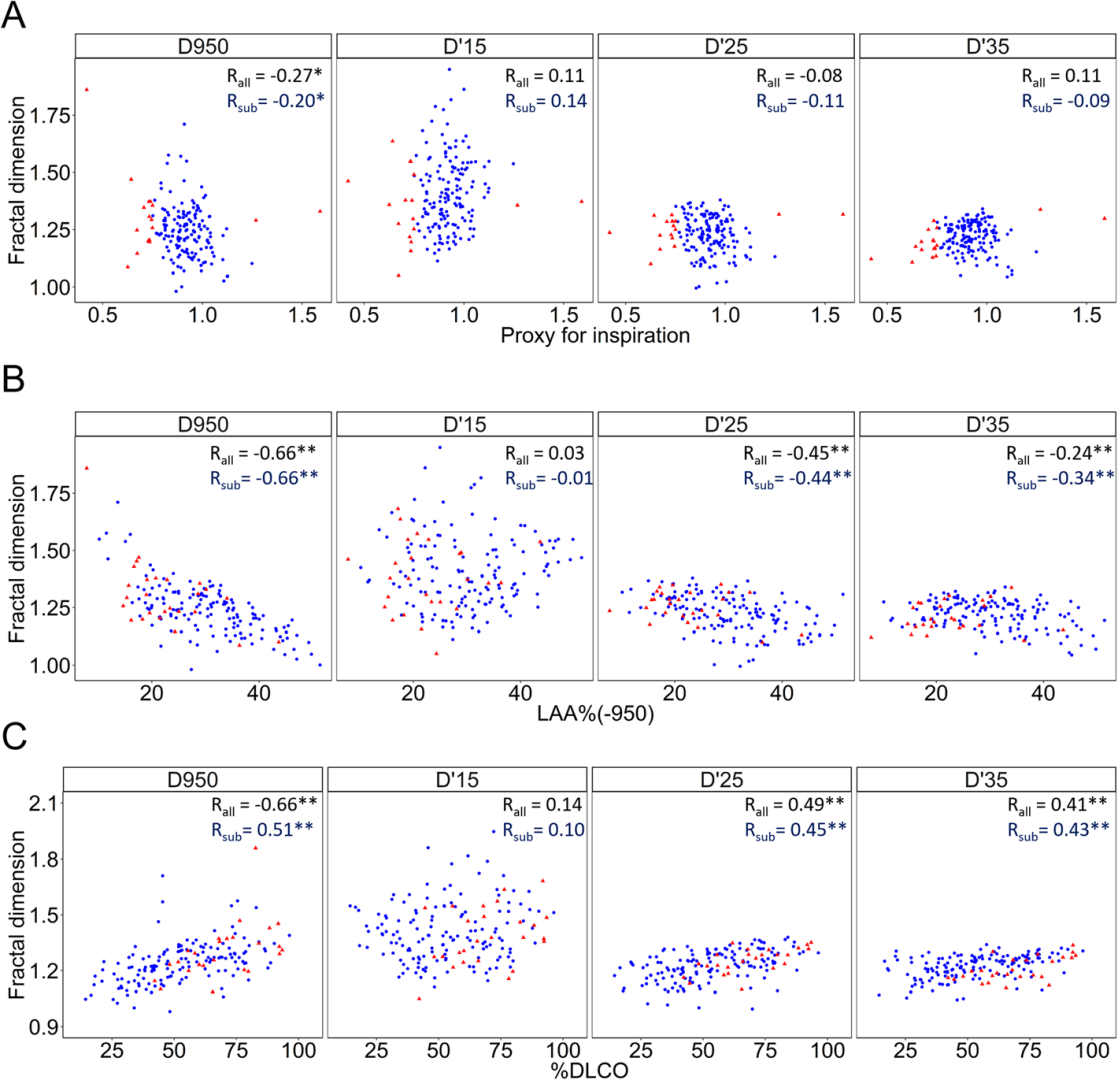
Associations of the fractal dimensions with a proxy for inspiration level at CT scan, LAA%, and diffusion capacity. **a** A proxy for inspiration indicates the degree of the inspiration level during a CT scan that was obtained as a ratio of CT-derived total lung volume (CT-TLV) to physiologically-measured total lung capacity (TLC). This proxy correlated with D based on a fixed threshold of − 950 HU (D950) but not with D based on a threshold of the 15th, 25th, and 35th percentiles of a CT density histogram. **b** An increase in percent low attenuation area (LAA%) and (**c**) a decrease in the diffusion capacity of carbon monoxide (%D_LCO_) were correlated with reductions in D950, D’25, and D’35, but not D’15. Blue dots indicate the 144 cases with better-quality scans defined as those with the proxies for inspiration > 0.8 and < 1.2. R_all_ and R_sub_ indicate coefficient correlations for the analysis of all scans (n = 170) and the subanalysis of the better-quality scans (*n* = 144), respectively. * *p* < 0.05 and ** *p* < 0.01

Figure 3a shows that the proxy for inspiration level during the CT scans was correlated with D950 but not with D’15, D’25, or D’35 in the analyses of all scans (*n* = 170, all dots) and the better-quality scans (*n* = 144, only blue dots). The LAA% and %DLCO (Fig. 3b and c) and the 15th percentile (Additional file 1: Figure S2) were correlated with D950, D’25, and D’35, but not with D’15. Table 2 summarizes correlation coefficients between CT indexes and pulmonary function in COPD in the analysis of 144 COPD patients with better-quality scan (the proxies for inspiration were > 0.8 and < 1.2). Multivariate regression analyses in Additional file 1: Table S1 showed that D’15, D’25, and D’35 were associated with %D_LCO_ and D_LCO_/V_A_ independently of LAA%, whereas D950 was not.

**Table 2:** Correlation coefficients between CT indexes and pulmonary function in COPD (*n* = 144)

	LAA%	D950	D’15	D’25	D’35
FEV_1_ (%predicted)	−0.45†	0.27†	0.02	0.21*	0.21*
RV/TLC	0.35†	−0.13	0.10	−0.02	0.03
D_LCO_ (%predicted)	−0.62†	0.48†	0.10	0.45†	0.43†
D_LCO_/V_A_	−0.71†	0.56†	0.14	0.47†	0.37†

Of 170 subjects enrolled in the present study, this analysis used 144 subjects in which the proxies for inspiration during CT scan were more than 0.8 and less than 1.2 because their CT scans were considered better for assessing structure-function relationships. *FEV*_*1*_ Forced expiratory volume in 1 s, *RV/TLC* Residual volume / total lung capacity, *D*_*LCO*_ Diffusion capacity, *V*_*A*_ Alveolar ventilation, *LAA%* Percent low attenuation area. D950, D’15, D’25, and D’35 are exponents that characterize a power law that governs the cumulative frequency size distribution of LAA clusters that are identified using a threshold of − 950 HU and the 15th, 25th, and 35th percentile of a CT density histogram, respectively. † *p* < 0.005 and * *p* < 0.05

### Analysis 2 (short-term longitudinal study using 3 CT scan within 1 year)

Additional file 1 Table S2 shows the demographics of 33 subjects used for short-term (< 1 year) longitudinal data analysis. Additional file 1: Figure S3 shows CV% of the fractal dimensions and CT-TLV, which were calculated from 3 values obtained at visits 1–3 for each of the 33 subjects with COPD. The CV% values for D950 and D’15 were less than 10% except for 1 subject and for 2 subjects, respectively, and the CV% for D’25, and D’35 were less than 10% in all subjects.

### Analysis 3 (long-term longitudinal study in current and former smokers)

Table 3 shows the demographics of 17 current and 42 former smokers included in long-term (> 5 years) longitudinal data analysis.

**Table 3:** Baseline characteristics of subjects in long-term longitudinal data

	Current smoker	Former smoker	*p*-Value
No. of subjects	17	42	
Follow-up period	6.2 ± 0.6	6.4 ± 0.7	0.22
Age	68 ± 6	67 ± 8	0.70
Sex (male: female)	17:0	42:0	
Body mass index	21 ± 3	22 ± 3	0.15
Pack-year	64 ± 31	62 ± 32	0.84
Pulmonary function			
FEV_1_ (% predicted)	61 ± 17	62 ± 19	0.81
TLC	5.7 ± 0.9	6.0 ± 0.9	0.27
RV/TLC (%)	43 ± 8	42 ± 8	0.46
D_LCO_ (% predicted)	56 ± 4	59 ± 3	0.55
D_LCO_/V_A_	3.0 ± 1	3.0 ± 1	0.97
CT			
LAA% (%)	24 ± 6	29 ± 8	0.04
D950	1.30 ± 0.08	1.25 ± 0.10	0.06
D’15	1.36 ± 0.13	1.36 ± 0.13	0.88
D’25	1.27 ± 0.02	1.23 ± 0.08	0.05
D’35	1.26 ± 0.04	1.23 ± 0.07	0.07
CT-TLV (L)	5.1 ± 0.9	5.4 ± 0.8	0.24
Proxy for inspiration	0.89 ± 0.07	0.91 ± 0.13	0.60

*FEV*_*1*_ Forced expiratory volume in 1 s, *RV/TLC* Residual volume / total lung capacity, *D*_*LCO*_ Diffusion capacity, *V*_*A*_ Alveolar ventilation. *LAA%* percent low attenuation area (LAA). D950, D’15, D’25, and D’35 are exponents that characterize a power law that governs the cumulative frequency size distribution of LAA clusters that are identified using a threshold of − 950 HU and the 15th, 25th, and 35th percentiles of a CT density histogram, respectively. A proxy for inspiration indicates the degree of the inspiration level during a CT scan that was obtained by dividing the total lung volume measured on CT (CT-TLV) by physiologically measured TLC

**Fig. 4:**
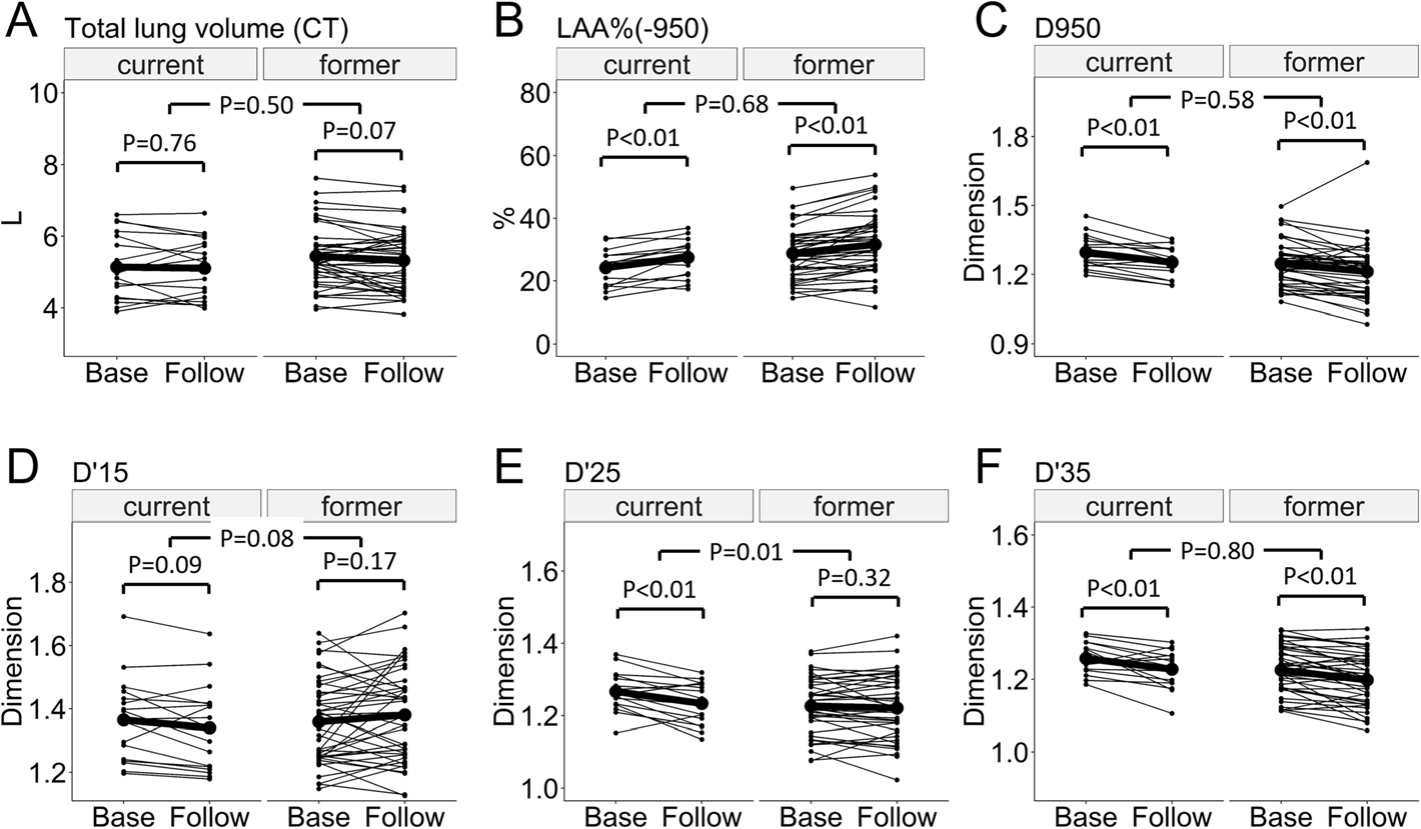
Long-term change in emphysema-related CT indexes in current and former smokers. **a** and **b** show long-term (at least 5 years) longitudinal changes in total lung volumes measured on CT, and LAA%(− 950) defined as percent low attenuation area less than − 950 HU in current and former smokers with COPD (*n* = 17 and 42, respectively). **c** D950, (**d**) D’15, (**e**) D’25, and (**f**) D’35 are exponents that characterize a power law that governs the cumulative frequency size distribution of LAA clusters that are identified using a threshold of − 950 HU and the 15th, 25th, and 35th percentiles of a CT density histogram, respectively. D950, D’15, D’25 and D’35, respectively. Base = baseline, Follow = follow-up

Figure 4 shows that in both current and former smokers, CT-TLV was not changed from the baseline to follow-up CT scans, while LAA% was increased and D950 and D’35 were decreased. In contrast, D’25 was reduced over time in current smokers but not in former smokers. This longitudinal change in D’25 was greater in current than former smokers, whereas the changes in CT-TLV, LAA%, D950, D’15, and D’35 did not differ between the two groups. Additional file 1: Figure S4 shows that the longitudinal changes in the 15th, 25th, and 35th percentiles did not differ between the two groups.

Table 4 shows the results of multivariate regression analysis to test whether current smoking status is associated with a longitudinal change in structural CT index after adjusting for change in CT- TLV and the baseline value of the CT index. The results show that current smoking status is independently associated with longitudinal change in D’25 and D’15 but not in LAA%, D950, and D’35.

**Table 4:** Multivariate regression analysis for longitudinal change in a CT index

Dependent variable	Independent variables		
	Current smoker (Ref. former smoker)	ΔCT-TLV	Baseline CT index
ΔLAA%	β* = 0.08 *p* = 0.68	β* = 0.72 *p* < 0.0005	β* = 0.14 *p* = 0.14
ΔD950	β* = − 0.16 *p* = 0.58	β* = − 0.34 *p* = 0.009	β* = 0.01 *p* = 0.92
ΔD’15	β* = − 0.59 *p* = 0.03	β* = 0.31 *p* = 0.01	β* = − 0.19 *p* = 0.12
ΔD’25	β* = − 0.71 *p* = 0.01	β* = 0.27 *p* = 0.03	β* = − 0.05 *p* = 0.68
ΔD’35	β* = 0.06 *p* = 0.84	β* = 0.41 *p* = 0.002	β* = − 0.07 *p* = 0.57

Five models were constructed. Each model included a longitudinal change in either LAA%, D950, D’15, D’25, or D’35 between baseline and follow-up CT scans (ΔLAA%, ΔD950, ΔD’15, ΔD’25, or ΔD’35) as a dependent variable. Smoking status (current vs former smoker), a longitudinal change in CT-derived total lung volume (ΔCT-TLV), and a corresponding baseline CT index (for example, the model for ΔLAA% used baseline LAA%) were included as independent variables for all models. D950, D’15, D’25, and D’35 are exponents that characterize a power law that governs the cumulative frequency size distribution of LAA clusters that are identified using a threshold of − 950 HU and the 15th, 25th, and 35th percentiles of a CT density histogram, respectively

## Discussion

This study proposed the novel fractal dimensions, D’15, D’25, and D’35, that characterize the cumulative size distribution of LAA clusters 3-dimensionally identified as regions below the 15th, 25th, and 35th percentiles of a CT density histogram, as an alternative to a conventional fixed threshold of − 950 HU [4, 21], and showed that D’25 more sensitively detects the longitudinal changes in emphysematous lungs than the conventional D950 and LAA%. The first analysis of cross-sectional data reveals that a reduction in D’25 correlates with impaired diffusion capacity and is robust against the inspiration level during a CT scan. The second analysis of short-term (< 1 year) longitudinal data confirms the good reproducibility of D’25 for multiple measurements. The third analysis of long-term (> 5 years) longitudinal data reveals that only D’25 allows the detection of statistically significant effects of continuous smoking on emphysema progression.

In the development and progression of emphysema, CT density is decreased, and LAA clusters appear and grow on CT scans. The authors of previous studies [4, 19] have calculated the fractal dimension regarding the size distribution of fixed-threshold-based LAA clusters and rejected a hypothesis that these LAA clusters are similar in size and distributed evenly in the entire lung. Alternatively, those studies have proposed that emphysematous change on CT is characterized by a few larger LAA clusters and many smaller clusters. The mechanism of this phenomenon was explained by the computer simulation showing that regions between pre-existing LAA clusters are more susceptible to destruction than other regions, which causes coalescence of neighboring LAA clusters and reduces the fractal dimension [4, 19, 21]. The present data extend this concept by showing that the cumulative size distribution of relatively lower density regions defined by the 15th, 25th, and 35th percentiles of a CT density histogram was also governed by the fractal property, suggesting that regions surrounded by relatively lower density regions are more susceptible to parenchymal destruction than those surrounded by normal density regions and that the pre-existing lower density regions tend to suffer from further reductions in CT density and form larger clusters of lower density regions.

The present data show complementary roles of D’25 and D950 in exploring morphological features of emphysematous lungs. Figure 2c and d show examples of 2 cases who similarly showed two giant LAA clusters and many small LAA clusters when using a fixed threshold of − 950 HU. The D950 values of these 2 cases were similar. However, when using the 25th percentile to define LAA clusters, the number of larger LAA clusters was greater in case 2 than in case 1, which made the size distribution of LAA clusters in these 2 cases different. These provided different D’25 values in these 2 cases, suggesting that D’25 provides additional information that is difficult to detect with D950. We postulate that D’25 is useful to compare the degree of the spatial heterogeneity in the distribution of relatively lower density regions for the entire lung between different individuals and between baseline and follow-up scans. In contrast, the threshold-based-indexes such as D950 and LAA% are useful to compare the sizes of emphysematous clusters locally.

Moreover, the data from the long-term (> 5 years) longitudinal analysis suggest that D’25 is more sensitive for detecting parenchymal destruction in a longitudinal study than the conventional CT indexes. Despite a relatively small sample size (*n* = 59), these data confirm the previous finding about the effect of continuous smoking on emphysema progression in a large observational study (*n* = 1928) by Coxson et al. [24], by demonstrating the significantly greater reduction in D’25 over time in current compared with former smokers. This greater change in D’25 in current smokers was further confirmed after adjusting the change in CT-TLV and the baseline D’25. In contrast, the analysis of D950 and LAA% was not able to detect significant differences between the two smoking groups, which might have been caused by the small sample size. Although a simple comparison of the longitudinal change in D’15 without the adjustments (Fig. 4d) did not show a statistically significant difference between the current and former smokers, the multivariate analysis showed that continuous smoking was associated with the longitudinal change in D’15, suggesting that in addition to D’25, D’15 might be useful in a longitudinal evaluation of emphysematous change. It should also be noted that continuous smoking might affect LAA% and the fractal dimensions as smoke increases inflammatory infiltrates and changes the CT density [30]. It is possible that the inflammatory infiltrate in current smokers mitigates an increase in LAA% and a decrease in the fractal dimensions due to parenchymal destruction, making it difficult to detect a statistically significant difference in their longitudinal changes between current and former smokers.

The long-term longitudinal data also show that LAA% is increased and D950 and D’35 are decreased in both current and former smokers, whereas D’25 is reduced in current smokers but is not altered in former smokers. Taken together, the present findings suggest that a combination of D’25 and the conventional structural index such as LAA% and D950 is more useful to evaluate longitudinal emphysema progression than the single use of either D’25, LAA%, or D950.

The univariate analysis showed that D950, D’25, and D’35 were correlated with LAA% and diffusion capacity assessed as %D_LCO_ and D_LCO_/V_A_. The result of the multivariate analysis shows that decreased D’25 was associated with impaired diffusion capacity independently of increased LAA%. We postulate that the diffusion capacity is determined not only by the overall emphysematous change in the whole lung but also by the spatial heterogeneity of parenchymal destruction, which is detected by D’25, but not by LAA%. The failure to detect an association of D950 with %D_LCO_ independently of LAA% in the multivariate analysis might have been caused by the close correlation between D950 and LAA%.

Since − 910 HU is also a fixed-threshold used to define LAA [12], we performed cross-sectional and longitudinal analyses using − 910 HU-based LAA% and D and confirmed similar results to those using − 950 HU-based LAA% and D (Additional file 1: Figure S5).

This study has some limitations. First, although CT densitometry is widely performed by using CT images reconstructed with a “standard” algorithm, we used a sharp algorithm in this study. Compared to smooth reconstruction, sharp reconstruction increases noise and thus LAA% [31, 32]. Nevertheless, this study is important because the presented method provides a new possibility for more sensitive assessment of parenchymal structure. In addition, since the CT data used here were generated using one CT scanner under one condition, the present results are very robust. Second, this study clearly shows that the new D’25 correlates well with the diffusion capacity, which correlates with the severity of emphysema on histology [16]. However, we have not investigated a direct correlation between D’25 and structural change on histology. Third, the subjects in the present study were mainly male. Because the severity and destruction of emphysema are different between males and females [33], further investigation is necessary to confirm whether the present findings can be applied to female subjects.

## Conclusion

In conclusion, this study has proposed a new fractal dimension, termed D’25, for the structural assessment of emphysematous lungs. D’25 is robust against variation in inspiration level during CT scans, and importantly has the potential for more sensitive detection of structural alterations over time than the conventional D950 and LAA%. Although these findings should be verified in a larger cohort, a combination of D’25 and fixed-threshold-based conventional indexes such as LAA% and D950 might be useful not only in observational studies about the natural course of the disease but also in pharmacological interventional studies aiming to evaluate the efficacies of new candidate drugs for preventing parenchymal destruction.

## Additional file

Additional file 1: Supplementary Methods, Figures, and Tables. (PDF 1246 kb)

## Abbreviations

CT: Computed tomography
CV%: Coefficient of variation percentage
D: Fractal dimension
D950, D’15, D’25, and D’35: Fractal dimensions that characterize a power law that governs the cumulative frequency size distribution of LAA clusters identified using thresholds of − 950 HU and the 15th, 25th, and 35th percentiles of a CT density histogram, respectively
D_LCO_: Diffusion capacity
FEV_1_: Forced expiratory volume in 1 s
HU: Hounsfield units
LAA: Low attenuation area
LAA%: Percent LAA
RV/TLC: Residual volume/total lung capacity
V_A_: Alveolar ventilation

## References

[1] Adeloye D, Chua S, Lee C, Basquill C, Papana A, Theodoratou E, Nair H, Gasevic D, Sridhar D, Campbell H (2015). Global and regional estimates of COPD prevalence: systematic review and meta-analysis. J Glob Health.

[2] Hogg JC (2004). Pathophysiology of airflow limitation in chronic obstructive pulmonary disease. Lancet.

[3] Nakano Y, Muro S, Sakai H, Hirai T, Chin K, Tsukino M, Nishimura K, Itoh H, Pare PD, Hogg JC (2000). Computed tomographic measurements of airway dimensions and emphysema in smokers. Correlation with lung function. Am J Respir Crit Care Med.

[4] Mishima M, Hirai T, Itoh H, Nakano Y, Sakai H, Muro S, Nishimura K, Oku Y, Chin K, Ohi M (1999). Complexity of terminal airspace geometry assessed by lung computed tomography in normal subjects and patients with chronic obstructive pulmonary disease. Proc Natl Acad Sci U S A.

[5] Han MK, Kazerooni EA, Lynch DA, Liu LX, Murray S, Curtis JL, Criner GJ, Kim V, Bowler RP, Hanania NA (2011). Chronic obstructive pulmonary disease exacerbations in the COPDGene study: associated radiologic phenotypes. Radiology.

[6] Haruna A, Muro S, Nakano Y, Ohara T, Hoshino Y, Ogawa E, Hirai T, Niimi A, Nishimura K, Chin K (2010). CT scan findings of emphysema predict mortality in COPD. Chest.

[7] Johannessen A, Skorge TD, Bottai M, Grydeland TB, Nilsen RM, Coxson H, Dirksen A, Omenaas E, Gulsvik A, Bakke P (2013). Mortality by level of emphysema and airway wall thickness. Am J Respir Crit Care Med.

[8] Regan EA, Lynch DA, Curran-Everett D, Curtis JL, Austin JH, Grenier PA, Kauczor HU, Bailey WC, DeMeo DL, Casaburi RH (2015). Clinical and radiologic disease in smokers with normal spirometry. JAMA Intern Med.

[9] Oelsner EC, Hoffman EA, Folsom AR, Carr JJ, Enright PL, Kawut SM, Kronmal R, Lederer D, Lima JA, Lovasi GS (2014). Association between emphysema-like lung on cardiac computed tomography and mortality in persons without airflow obstruction: a cohort study. Ann Intern Med.

[10] Lynch DA, Austin JH, Hogg JC, Grenier PA, Kauczor HU, Bankier AA, Barr RG, Colby TV, Galvin JR, Gevenois PA, et al. CT-definable subtypes of chronic obstructive pulmonary disease: a statement of the Fleischner society. Radiology. 2015;277(1):192-205. 10.1148/radiol.2015141579 PMC4613878 25961632

[11] Madani A, Zanen J, de Maertelaer V, Gevenois PA (2006). Pulmonary emphysema: objective quantification at multi-detector row CT--comparison with macroscopic and microscopic morphometry. Radiology.

[12] Parr DG, Sevenoaks M, Deng C, Stoel BC, Stockley RA (2008). Detection of emphysema progression in alpha 1-antitrypsin deficiency using CT densitometry; methodological advances. Respir Res.

[13] Parr DG, Stoel BC, Stolk J, Stockley RA (2006). Validation of computed tomographic lung densitometry for monitoring emphysema in alpha1-antitrypsin deficiency. Thorax.

[14] Muller NL, Staples CA, Miller RR, Abboud RT (1988). “Density mask”. An objective method to quantitate emphysema using computed tomography. Chest.

[15] Gevenois PA, De Vuyst P, de Maertelaer V, Zanen J, Jacobovitz D, Cosio MG, Yernault JC (1996). Comparison of computed density and microscopic morphometry in pulmonary emphysema. Am J Respir Crit Care Med.

[16] Gould GA, MacNee W, McLean A, Warren PM, Redpath A, Best JJ, Lamb D, Flenley DC (1988). CT measurements of lung density in life can quantitate distal airspace enlargement--an essential defining feature of human emphysema. Am Rev Respir Dis.

[17] Mandelbrot B (1977). The fractal geometry of nature.

[18] Suki B (2002). Fluctuations and power laws in pulmonary physiology. Am J Respir Crit Care Med.

[19] Suki B, Lutchen KR, Ingenito EP (2003). On the progressive nature of emphysema: roles of proteases, inflammation, and mechanical forces. Am J Respir Crit Care Med.

[20] Coxson HO, Whittall KP, Nakano Y, Rogers RM, Sciurba FC, Keenan RJ, Hogg JC (2003). Selection of patients for lung volume reduction surgery using a power law analysis of the computed tomographic scan. Thorax.

[21] Tanabe N, Muro S, Hirai T, Oguma T, Terada K, Marumo S, Kinose D, Ogawa E, Hoshino Y, Mishima M (2011). Impact of exacerbations on emphysema progression in chronic obstructive pulmonary disease. Am J Respir Crit Care Med.

[22] Tanabe N, Muro S, Sato S, Tanaka S, Oguma T, Kiyokawa H, Takahashi T, Kinose D, Hoshino Y, Kubo T (2012). Longitudinal study of spatially heterogeneous emphysema progression in current smokers with chronic obstructive pulmonary disease. PLoS One.

[23] Dirksen A, Friis M, Olesen KP, Skovgaard LT, Sorensen K (1997). Progress of emphysema in severe alpha 1-antitrypsin deficiency as assessed by annual CT. Acta Radiol.

[24] Coxson HO, Dirksen A, Edwards LD, Yates JC, Agusti A, Bakke P, Calverley PM, Celli B, Crim C, Duvoix A (2013). The presence and progression of emphysema in COPD as determined by CT scanning and biomarker expression: a prospective analysis from the ECLIPSE study. Lancet Respir Med.

[25] Terada K, Muro S, Sato S, Ohara T, Haruna A, Marumo S, Kinose D, Ogawa E, Hoshino Y, Niimi A (2008). Impact of gastro-oesophageal reflux disease symptoms on COPD exacerbation. Thorax.

[26] Tanabe N, Vasilescu DM, Kirby M, Coxson HO, Verleden SE, Vanaudenaerde BM, Kinose D, Nakano Y, Pare PD, Hogg JC: Analysis of airway pathology in COPD using a combination of computed tomography, micro-computed tomography and histology. Eur Respir J 2018, 51(2). 10.1183/13993003.01245-2017 PMC6691959 29444912

[27] Kirby M, Tanabe N, Tan WC, Zhou G, Obeidat M, Hague CJ, Leipsic J, Bourbeau J, Sin DD, Hogg JC (2018). Total airway count on computed tomography and the risk of chronic obstructive pulmonary disease progression. Findings from a population-based study. Am J Respir Crit Care Med.

[28] Tanabe N, Muro S, Tanaka S, Sato S, Oguma T, Kiyokawa H, Takahashi T, Kinose D, Hoshino Y, Kubo T (2012). Emphysema distribution and annual changes in pulmonary function in male patients with chronic obstructive pulmonary disease. Respir Res.

[29] R Core Team: R: A Language and Environment for Statistical Computing. 2015. http://www.r-project.org/.

[30] Ashraf H, Lo P, Shaker SB, de Bruijne M, Dirksen A, Tonnesen P, Dahlback M, Pedersen JH (2011). Short-term effect of changes in smoking behaviour on emphysema quantification by CT. Thorax.

[31] Sieren JP, Newell JD, Barr RG, Bleecker ER, Burnette N, Carretta EE, Couper D, Goldin J, Guo J, Han MK (2016). SPIROMICS protocol for multicenter quantitative computed tomography to phenotype the lungs. Am J Respir Crit Care Med.

[32] Gierada DS, Bierhals AJ, Choong CK, Bartel ST, Ritter JH, Das NA, Hong C, Pilgram TK, Bae KT, Whiting BR (2010). Effects of CT section thickness and reconstruction kernel on emphysema quantification relationship to the magnitude of the CT emphysema index. Acad Radiol.

[33] Martinez FJ, Curtis JL, Sciurba F, Mumford J, Giardino ND, Weinmann G, Kazerooni E, Murray S, Criner GJ, Sin DD (2007). Sex differences in severe pulmonary emphysema. Am J Respir Crit Care Med.

