# Supplementary material for: Fractal analysis of low attenuation clusters on computed tomography in chronic obstructive pulmonary disease
Source: BMC Pulm Med. 2018 Aug 29;18:144. doi: 10.1186/s12890-018-0714-5 (PMC6116481; doi:10.1186/s12890-018-0714-5)
Supplement: Supplementary file 1 — Supplementary Methods, Figures, and Tables. (PDF 1246 kb) [file 12890_2018_714_MOESM1_ESM.pdf]

Online Supplement

Fractal analysis of low attenuation clusters on computed tomography in chronic obstructive pulmonary disease

Naoya Tanabe, Shigeo Muro, Susumu Sato, Tsuyoshi Oguma, Atsuyasu Sato, Toyohiro Hirai

Department of Respiratory Medicine, Graduate School of Medicine, Kyoto University, Kyoto Japan

List:

- ✓ **Methods. Specific inclusion and exclusion criteria for subjects in 3 analyses**

- ✓ **Figure S1. Examples of low attenuation cluster analysis using either the threshold of a fixed CT density or that of the 25th percentile of the CT density histogram**
- ✓ **Figure S2. Associations of the fractal dimensions with the 15th percentile of a CT density histogram**
- ✓ **Figure S3. Variability in computed tomography emphysematous indexes in short-term longitudinal analysis**
- ✓ **Figure S4. Long-term changes in computed tomography emphysema indexes in current and former smokers**
- ✓ **Figure S5. Low attenuation cluster analysis using a fixed-threshold of -910 HU**

- ✓ **Table S1. Multivariate linear regression analysis to identify the relative contribution of LAA% and fractal dimension to diffusion capacity (n=144)**
- ✓ **Table S2. Demographics of the subjects for short-term longitudinal analysis**

Supplementary Methods

Specific inclusion and exclusion criteria for subjects in 3 analyses

(1) Cross-sectional data analysis

The first analysis included 170 subjects with COPD who underwent chest CT scans and pulmonary function tests during non-exacerbating periods between April 2010 and March 2014. The specific exclusion criteria for this first analysis were abnormal shadows on chest CT scan such as fibrotic lesion.

(2) Short-term longitudinal data analysis

The second analysis included 33 subjects who underwent 3 CT scans within 1 year because they required close follow-up of small abnormal shadows other than emphysema (short-term longitudinal study). The specific exclusion criteria for this second analysis were any clinically unstable conditions during the one year, including exacerbation of COPD, pneumonia, and cardiac failure.

(3) Long-term longitudinal data analysis

The third analysis initially included 60 subjects who were also enrolled in the same observational study and were followed-up for at least 5 years (long-term longitudinal study). The specific inclusion criteria for this third analysis were (1) availability of the baseline and >5-year apart CT scans, (2) no change in smoking status during the follow-up, and (3) absence of abnormal shadow other than emphysematous change on the baseline and follow-up CT scans. In addition, to exclude subjects whose quality of CT scan was poor due to inappropriate breath holding at scan, a more than 20% change in CT-measured total lung volume (CT-TLV) from the baseline to the follow-up scans was also used as an exclusion criterion. Consequently, 1 subject who showed a 32% reduction in CT-TLV at the follow-up scan was excluded, and 59 subjects were used in the third analysis.

Supplementary Figures

Figure S1. Examples of low attenuation cluster analysis using either the threshold of a fixed CT density or that of the 25th percentile of the CT density histogram

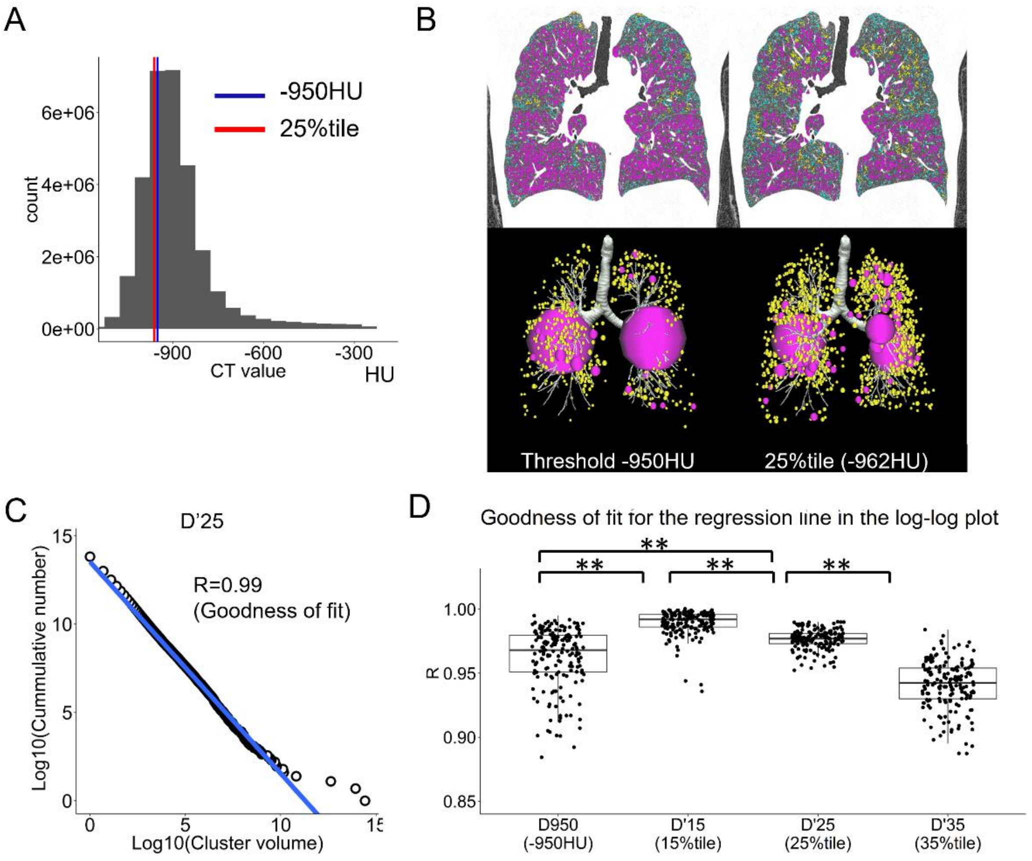

(A) An example of a CT density histogram. Low attenuation areas (LAAs) have been defined using a threshold of either -950 Hounsfield Units (HU) or the 25th percentiles of the histogram, which are indicated with blue and red vertical lines, respectively. (B) LAA clusters based on a threshold of the fixed -950 HU and the 25th of the histogram in the 3D representation were expressed as spheres with volumes equivalent to those of actual clusters. Pink, yellow, and blue regions indicate large (>500 mm^3), moderate (>50 and <499 mm^3), and small (<50 mm^3) clusters. Small clusters were omitted in the 3D representation. (C) The log-transformed volumes of the LAA clusters and the log-transformed cumulative counts of the clusters larger than the given volume were plotted. This plot was well characterized by linear regression, suggesting that the cumulative number Y of the clusters larger than the volume X could be described with the following formula: $Y=K \times X^{-D}$, also known as the fractal property. The D value is fractal dimension that is obtained as an absolute slope of the regression line on the log-log plot. D'25 indicates the fractal D characterizing LAA clusters based on the 25th percentile. (D) The goodness of fit for the regression line on the log-log plot. ** p<0.01 after Holm correction.

Figure S2. Associations of the fractal dimensions with the 15th percentile of a CT density histogram

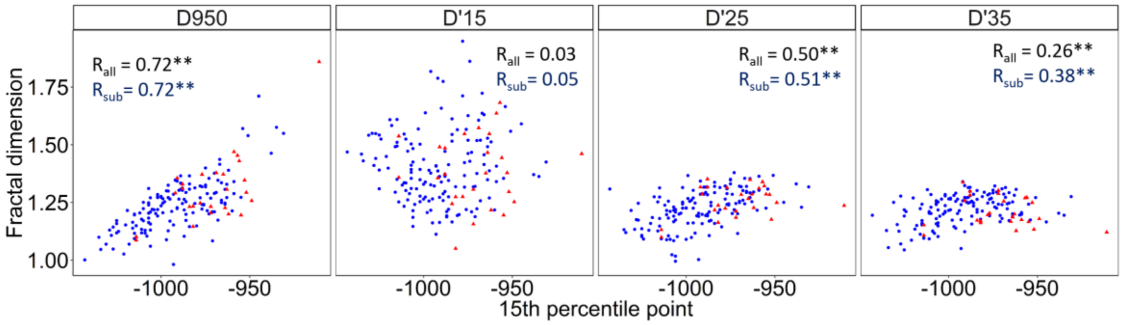

D950 indicates D based on a fixed threshold of -950 HU (D950). D'15, D'25, and D'35 indicate D based on a threshold of the 15th, 25th, and 35th percentiles of a CT density histogram, respectively. A proxy for inspiration during a CT scan was obtained as a ratio of CT-derived total lung volume (CT-TLV) to physiologically-measured total lung capacity (TLC). Blue dots indicate 144 cases with the better-quality scans defined as those with the proxy for inspiration >0.8 and <1.2. D950, D'25, and D'35, but not D'15 were correlated with the 15th percentiles of a CT density histogram. R_{all} and R_{sub} indicate coefficient correlations for analysis of all scans (n=170) and sub-analysis of the better-quality scans (n=144), respectively. * p<0.05 and ** p<0.01.

Figure S3. Variability in computed tomography emphysematous indexes in short-term longitudinal analysis

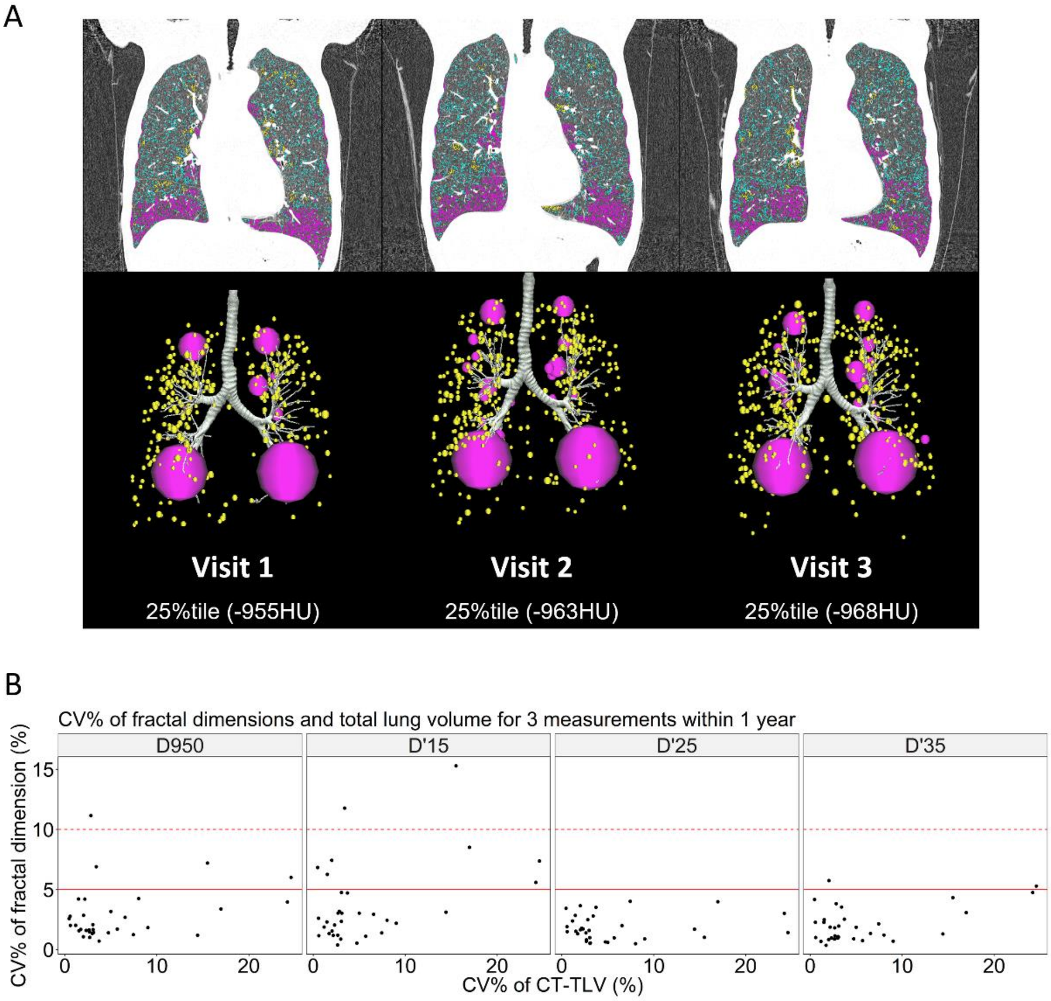

(A) An example of low attenuation area (LAA) clusters based on the 25th percentile of a CT density histogram in the analysis of the longitudinal data. LAA clusters based on a threshold of the fixed -950 HU and the 25th of the histogram in the 3D representation were expressed as spheres with volumes equivalent to those of actual clusters. Pink, yellow, and blue regions indicate large (>500 mm^3), moderate (>50 and <499 mm^3), and small (<50 mm^3) clusters. Small clusters were omitted in the 3D representation. (B) The coefficient of variations (CV%) of D950, D'15, D'25 and D'35 were compared to the CV% of CT-derived total lung volume (CT-TLV) obtained from 3 measurements within a year in stable subjects with COPD. the CV% values for D950, D'15, D'25, and D'35 were 10% or lower in more than 90% subjects regardless of the variation of CT-TLV.

Figure S4. Long-term longitudinal changes in the 15th, 25th, and 35th percentile points of a CT density histogram in current and former smokers

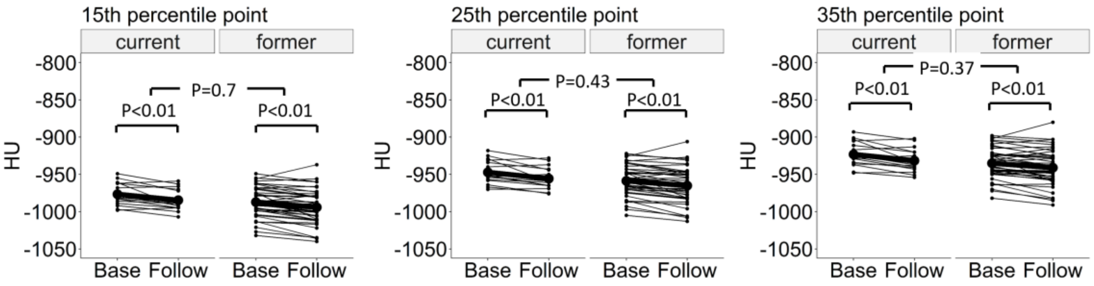

Long-term (at least 5 years) longitudinal changes in the 15th, 25th, and 35th percentile points of a CT density histogram in 17 current and 42 former smokers with COPD. Base = baseline, Follow = follow-up.

Figure S5 Low attenuation cluster analysis using a fixed threshold of -910 HU

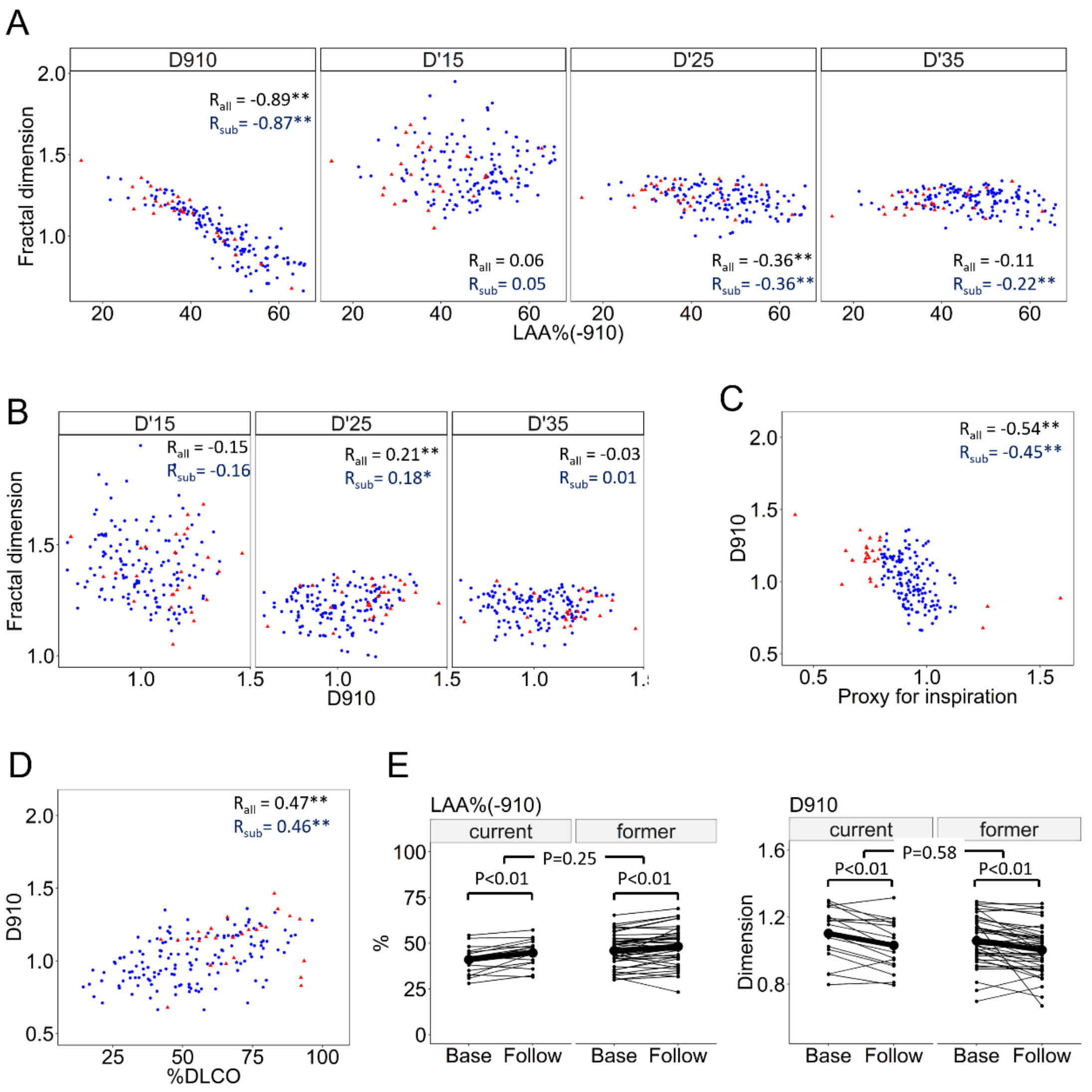

D910 indicates D based on a fixed threshold of -910 HU. D'15, D'25, and D'35 indicate D based on a threshold of the 15th, 25th, and 35th percentiles of a CT density histogram, respectively. A proxy for inspiration during a CT scan was obtained as a ratio of CT-derived total lung volume (CT-TLV) to physiologically-measured total lung capacity (TLC). Blue dots indicate 144 cases with the better-quality scans defined as those with the proxy for inspiration >0.8 and <1.2. (A) percent low attenuation area <-910 HU [LAA%(-910)] was correlated with D910, D'25, and D'35, but not D'15. (B) D910 was correlated with D'25. The proxy for inspiration (C) and diffusion capacity assessed as %D_{LCO} (D) were correlated with D910. R_{all} and R_{sub} indicate coefficient correlations for analysis of all scans (n=170) and sub-analysis of the better-quality scans (n=144), respectively. (E) Long-term (at least 5 years) longitudinal changes in LAA%(-910) and D910 in 17 current and 42 former smokers with COPD. * p<0.05 and ** p<0.01.

Supplementary Tables

Table S1. Multivariate linear regression analysis to identify the relative contribution of LAA% and fractal dimension to diffusion capacity (n=144)

	Dependent variable	Independent Variables	β*	P value
Model 1	%DLCO	LAA%	-0.49	<0.0005
		D950	0.12	0.17
Model 2	%DLCO	LAA%	-0.57	<0.0005
		D'15	0.15	0.02
Model 3	%DLCO	LAA%	-0.44	<0.0005
		D'25	0.26	<0.0005
Model 4	%DLCO	LAA%	-0.45	<0.0005
		D'35	0.27	<0.0005
Model 5	D_{LCO}/V_A	LAA%	-0.67	<0.0005
		D950	0.13	0.08
Model 6	D_{LCO}/V_A	LAA%	-0.76	<0.0005
		D'15	0.17	0.003
Model 7	D_{LCO}/V_A	LAA%	-0.65	<0.0005
		D'25	0.21	0.0008
Model 8	D_{LCO}/V_A	LAA%	-0.71	<0.0005
		D'35	0.12	0.04

Each model included age, sex, smoking pack-years, body mass index, and the proxy for inspiration level during a CT scan as independent variables in addition to LAA% and one of fractal dimension. D950, D'15, D'25, and D'35 are exponents that characterize a power law that governs the cumulative frequency size distribution of LAA clusters that are identified using a threshold of -950 HU and the 15th, 25th, and 35th percentile of a CT density histogram, respectively.

Table S2. Demographics of the subjects for short-term longitudinal analysis

No. of subjects	33
Age	70 ± 7
Sex (male:female)	32: 1
Body mass index	
Pack-year	77 ± 40
Pulmonary function	
FEV_1 (% predicted)	56 ± 19
RV/TLC (%)	44 ± 7†
D_{LCO} (% predicted)	51 ± 20†
D_{LCO}/V_A	2.7 ± 1.1†
CT	
LAA% (%)	27 ± 10
Total lung volume (L)	5.2 ± 1.1

Data are expressed as the mean ± SD. FEV_1 = Forced expiratory volume in 1 second, RV/TLC = Residual volume / total lung capacity, D_{LCO} = Diffusion capacity, V_A= Alveolar ventilation, LAA% = Percent low attenuation area. † RV/TLC, D_{LCO}, and D_{LCO}/V_A were measured in 31 out of 33 patients.
